# A case of pulmonary carcinoid tumor with concomitant tuberculosis

**DOI:** 10.4103/0970-2113.56349

**Published:** 2009

**Authors:** Ramakant Dixit, Rakesh Gupta, Ajay Yadav, A. R. Paramez, Gautam Sen, Sidharth Sharma

**Affiliations:** *Department of Pulmonary Medicine, J. L. N. Medical College, Ajmer, India*; 1 *Department of Pathology, J. L. N. Medical College, Ajmer, India*; 2 *Department of Thoracic Surgery, J. L. N. Medical College, Ajmer India*; 3 *Department of SDM Hospital, Jaipur, India*

**Keywords:** Carcinoid tumor, pulmonary tuberculosis, coexistence

## Abstract

The simultaneous occurrence of pulmonary carcinoid tumor and tuberculosis is very rare. A case of pulmonary carcinoid tumor is described in a 35-year-old male patient who had concomitant ipsilateral pulmonary tuberculosis. The importance of dual pathological diagnosis in clinical practice is also emphasized.

## INTRODUCTION

Pulmonary carcinoid is an uncommon tumor that constitutes only 0.5% to 2.5% of all pulmonary neoplasms. It is a low-grade malignant neoplasm believed to be derived from surface or glandular epithelium of the conducting or transitional airways.[1] Most of these tumors are located centrally and produce symptoms and signs of bronchial obstruction, including cough, fever, chest pain and often a localized wheeze. Hemoptysis is present in approximately 50%, reflecting both their central origin and hypervascularity.[2]

The simultaneous occurrence of pulmonary carcinoid tumor and tuberculosis is very unusual. Although all histological types of lung cancer can coexist with pulmonary tuberculosis,[3] only few coexisting cases of bronchial carcinoid tumor and pulmonary tuberculosis have been reported in the literature.[4,5] The present communication describe pulmonary carcinoid tumor in a young male patient who had concomitant pulmonary tuberculosis in the same lung. To the best of author's knowledge, such presentation has not been described previously in Indian literature.

## CASE REPORT

A 35-year-old man presented with history of right-sided pleuritic chest pain and low-grade fever for last one month. There were no other complaints. He also had history of hemoptysis and cough five months back for which he was investigated and found to have some pulmonary abnormality on chest X-ray. Although sputum status for acid-fast bacilli was not available in previous records, the tuberculin reaction was strongly positive (18-mm induration with blister formation). He received daily antituberculosis therapy with streptomycin, isoniazid, rifampicin and ethambutol for two months before presenting to us. He was a graduate and denied any history of addiction.

On examination patient had normal body mass index and no significant findings on routine physical examination. Respiratory system examination revealed altered percussion note and diminished intensity of breath sounds over the right infrascapular area. Other systemic examination was unremarkable.

**Figure 1:**
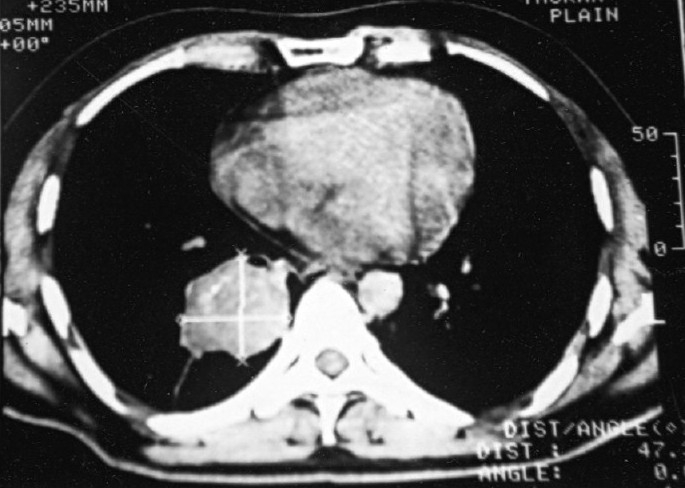
CT scan thorax showing soft-tissue density mass (carcinoid tumor) at medial segment of the right lower lobe

**Figure 2:**
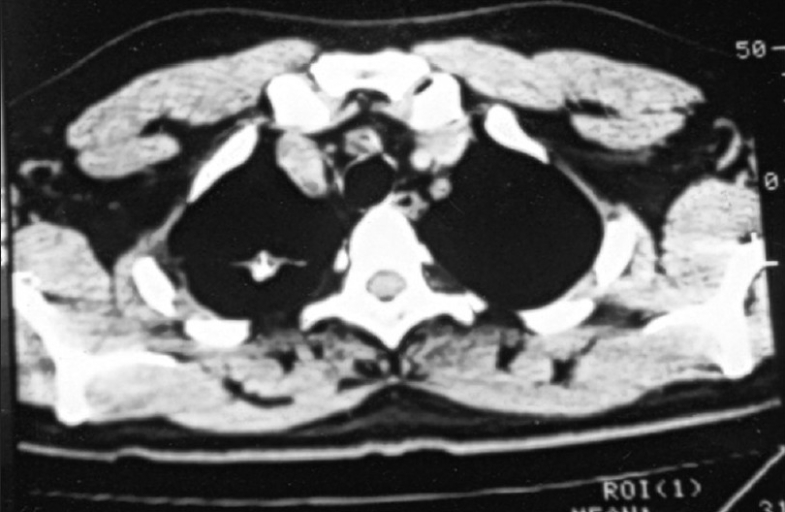
CT scan thorax showing a nodular lesion (tuberculosis) at the right upper lobe

The patient's investigations revealed normal hemoglobin, blood counts, bleeding profile and other organ functions. Chest X-ray showed a homogenous opacity without airbronchogram in the right lower zone. His sputum was negative for acid-fast bacilli. Spirometry revealed moderate restrictive pattern (one-second forced expiratory volume [FEV_1_] 62%, forced vital capacity [FVC] 65% of predicted and FEV_1_/FVC ratio 80.7%). Ultrasonography of abdomen was reported to be normal. On CT scan of thorax, there was a well-defined soft-tissue density lesion in relation with the right lower lobe bronchus, having few areas of calcification and narrowing the lumen. A linear soft-tissue density band was extending from the lesion to the pleura, suggesting a collapse segment. A small tissue-density lesion was also evident in the right upper lobe [Figures 1 and 2].

**Figure 3:**
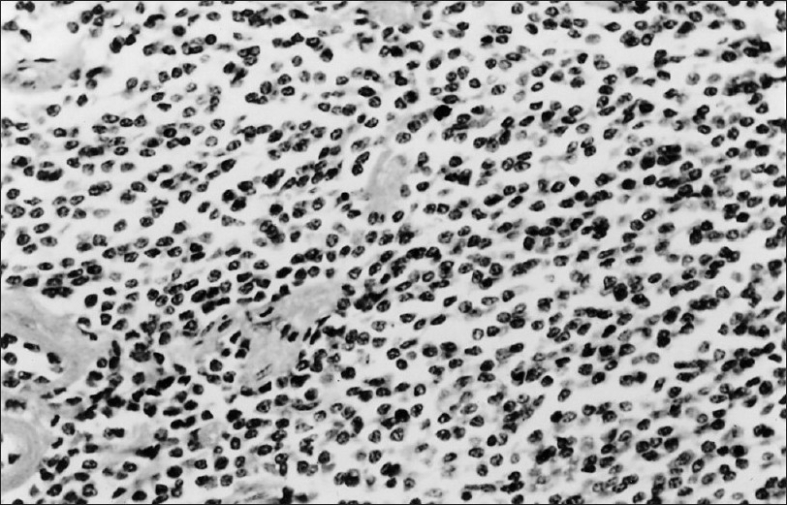
Photomicrograph of carcinoid tumor lesion from the lower lobe mass showing monomorphic tumor cells forming small nests, rosette-like arrangements and cords with chromatin showing salt and pepper, stippled appearance (H and E, ×400)

**Figure 4:**
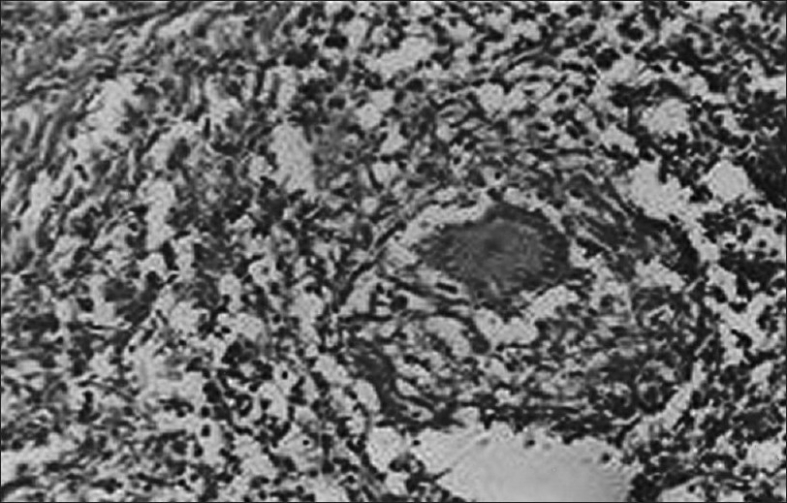
Photomicrograph of tuberculosis lesion from upper lobe nodule showing epitheleoid cell granuloma with langerhans-type giant cells (H and E, ×400)

For tissue diagnosis, patient underwent fiberoptic bronchoscopy that revealed an endobronchial rounded vascular mass at the right lower lobe medial segment. This mass was blocking the lumen and bleeding when touched. The biopsies and bronchial washings could not be taken in view of profuse bleeding. A possibility of malignant lesion at the right lower lobe with an ipsilateral upper lobe metastasis was considered, and the patient was operated for right-sided pneumonectomy. Gross examination of the right lung revealed a tumor measuring  4 × 3 × 2 cm in the right lower lobe bronchus. The cut surface appeared grayish and it completely obstructed the lumen. In the upper lobe, a small nodular area measuring 0.3 × 0.2 × 0.1 cm with grayish cut surface was also sampled. Histopathological report of these lesions was as follows: (i) right lower lobe tumor: Carcinoid tumor [Figure 3]; (ii) right upper lobe pulmonary nodule: Tuberculosis [Figure 4]; and (iii) lymph node: Reactive hyperplasia.

The postoperative period was uneventful and he received 5-flourouracil 750 mg intravenously every 15 days for 3 months along with antituberculosis treatment for 1 year (2 HRZE/10 HR). One year later, his chest X-ray showed right opaque hemithorax with normal left lung fields. On bronchoscopy, the right bronchial stump was well healed. The CT scan thorax revealed no evidence of tumor recurrence, lymphadenopathy or focal pulmonary parenchymal lesions. The abdominal CT scan also ruled out any hepatic lesion, retroperitoneal lymphadenopathy or ascites. The patient did well in the following six years and then was lost to follow-up.

## DISCUSSION

Carcinoid tumors are neuroendocrine tumors arising from kultchitzsky cells. They can be central or peripheral, and on the basis of histological and cytological features, can be divided in two fairly distinct clinicopathologic types, that is, typical and atypical.[6] Both variants can be asymptomatic but central carcinoid often present with recurrent pneumonias or hemoptysis. The carcinoid syndrome occurs infrequently with an incidence as low as 0% to 3% and presentation includes flushing, wheezing, anxiety, vomiting and hypotension due to production of 5-hydroxitryptamine, bradykinin, prostaglandin, etc. This syndrome always reflects metastasis of carcinoid tumor, usually to the liver. Other rare manifestations includes Zollinger-Ellison syndrome, hyperinsulinemia and association with multiple endocrine neoplasia type I.[7] None of these features were present in our patient who presented with only pleuritic chest pain that was possibly due to postobstructive pneumonitis process.

The radiological findings in central tumors are pneumonitis, atelectasis and bronchiectasis. Nonobstructing central and peripheral tumor may appear as solitary pulmonary nodule. Computed tomographic scanning is useful in identifying endobronchial lesion as well as lymph node enlargement.[1] The CT scan findings in our case were a mass lesion at the right lower lobe and a nodule at the right upper lobe, suggesting metastasis; however, these two lesions were totally different on histopathological examination.

The coexistence of lung cancer and pulmonary tuberculosis is well known; however, the coexistence of bronchial carcinoid and pulmonary tuberculosis has been rarely reported in the English literature, possibly because the pulmonary carcinoid tumors are rare tumors. Agaev[4] reported that among 37 patients with bronchial carcinoid tumors, 9 patients had coexistence of pulmonary tuberculosis. Yilmaz and coworkers[5] reported only single case among 24 cases of bronchial carcinoid tumor having tuberculosis in the same lobe during 8-year study period. In our case, the carcinoid tumor was located at the lower lobe and tuberculous lesion at the upper lobe in the same lung with no suspicion of two different diseases before histopathological examination of the resected lung. There is a close relationship between the location of these two diseases and the time required for diagnosis. When tumor and tuberculosis are located in different lobes, the time required for diagnosis is shorter. When two diseases are located in the same lobe, the diagnosis time may be longer because one can mask the other condition.[5]

The pulmonary tumorlet are localized, minute lesions resembling carcinoid tumor caused by nodular proliferation of neuroendocrine cells of the respiratory tract extending beyond the epithelium into the adjacent wall or lung parenchyma. These are small usually microscopic, tumor-like lesions mostly found in damaged lung tissue, for example, bronchiectasis, chronic abscess, tuberculosis and sometime even carcinoid tumor itself.[1] It is interesting to note that pulmonary tumorlet so-called “microscopic carcinoid tumor” is also known to occur in association with fibrous tuberculous lesions and at times the demarcation between pulmonary tumorlet and carcinoid is very little when a tumorlet nodule greater than 5 mm may be considered a small carcinoid tumor.[1] There is also a report of pulmonary tumorlet with caseous granuloma associated with atypical mycobacterium.[8] In the present case, patient had concomitant carcinoid tumor lesion and tuberculosis on histological examination of the resected lung and this association seems coincidental one.

## CONCLUSION

The coexistence of pulmonary carcinoid tumor and pulmonary tuberculosis is very rare. When two diseases are located in the same lung, the diagnosis may be difficult at times until surgical intervention as one lesion can mask the other. The clinicians must be fully aware that two different pathological processes may coexist in the same patient, and therefore in nonresponding tuberculosis cases, dual pathology or alternative diagnosis must be considered apart from other causes such as drug resistance or paradoxical response and should be thoroughly investigated accordingly.
